# GBV-Net: Hierarchical Fusion of Facial Expressions and Physiological Signals for Multimodal Emotion Recognition

**DOI:** 10.3390/s25206397

**Published:** 2025-10-16

**Authors:** Jiling Yu, Yandong Ru, Bangjun Lei, Hongming Chen

**Affiliations:** School of Information Engineering, Zhejiang Ocean University, Zhoushan 316022, China; yujiling@zjou.edu.cn (J.Y.); bangjunlei@zjou.edu.cn (B.L.); 2022048@zjou.edu.cn (H.C.)

**Keywords:** GBV-Net, facial expressions, physiological signals, multimodal fusion, emotion recognition

## Abstract

A core challenge in multimodal emotion recognition lies in the precise capture of the inherent multimodal interactive nature of human emotions. Addressing the limitation of existing methods, which often process visual signals (facial expressions) and physiological signals (EEG, ECG, EOG, and GSR) in isolation and thus fail to exploit their complementary strengths effectively, this paper presents a new multimodal emotion recognition framework called the Gated Biological Visual Network (GBV-Net). This framework enhances emotion recognition accuracy through deep synergistic fusion of facial expressions and physiological signals. GBV-Net integrates three core modules: (1) a facial feature extractor based on a modified ConvNeXt V2 architecture incorporating lightweight Transformers, specifically designed to capture subtle spatio-temporal dynamics in facial expressions; (2) a hybrid physiological feature extractor combining 1D convolutions, Temporal Convolutional Networks (TCNs), and convolutional self-attention mechanisms, adept at modeling local patterns and long-range temporal dependencies in physiological signals; and (3) an enhanced gated attention fusion module capable of adaptively learning inter-modal weights to achieve dynamic, synergistic integration at the feature level. A thorough investigation of the publicly accessible DEAP and MAHNOB-HCI datasets reveals that GBV-Net surpasses contemporary methods. Specifically, on the DEAP dataset, the model attained classification accuracies of 95.10% for Valence and 95.65% for Arousal, with F1-scores of 95.52% and 96.35%, respectively. On MAHNOB-HCI, the accuracies achieved were 97.28% for Valence and 97.73% for Arousal, with F1-scores of 97.50% and 97.74%, respectively. These experimental findings substantiate that GBV-Net effectively captures deep-level interactive information between multimodal signals, thereby improving emotion recognition accuracy.

## 1. Introduction

Emotion recognition, a key technology in human–computer interaction (HCI) and a core application of artificial intelligence (AI) [1], allows computer systems to accurately perceive human emotional states in real time. This capability enables adaptive HCI models, forming the foundation for natural user experiences. Significant progress in emotion recognition has led to its widespread use in diverse applications, including driver rage detection [2], specialized patient care [3], and adolescent mental health assessment [4].

While unimodal recognition using facial expressions or physiological signals is well-established, emotion as a complex psychophysiological phenomenon often lacks robustness when analyzed through single modalities or even fused physiological signals alone [5]. Current research primarily uses visual data (e.g., facial images and video) and physiological data (e.g., EEG, ECG, and GSR) [6]. Facial expressions, observable emotional cues, are easily captured via cameras, with features extractable by handcrafted or deep learning methods. Physiological signals, originating from nervous system activity, are less susceptible to conscious control and may better reflect genuine emotional states. However, physiological signal acquisition faces challenges like hardware heterogeneity and specialized preprocessing needs, limiting deep learning exploration for this modality. Fusing visual and physiological data is essential for more accurate emotion recognition. Yet, current models predominantly rely on simplistic fusion strategies such as early feature concatenation or linear weighting. These approaches fail to capture the complex, nonlinear interactions between modalities, treat each modality as independent and static, and cannot adaptively adjust the contribution of each modality based on input-specific reliability. This results in suboptimal utilization of complementary information and limited performance gains, especially when one modality is noisy or ambiguous. Furthermore, both subtle facial changes and rhythmic physiological fluctuations are inherently temporal processes. Existing research, to our knowledge, lacks systematic modeling of this crucial temporal dynamic.

Therefore, this study focuses on improving feature extraction methods for visual (facial images) and physiological signals, while exploring more effective multimodal feature fusion strategies, aiming to improve the performance of affective computing systems in terms of both prediction reliability and generalization capability. Specifically, we design a computationally efficient, ConvNeXt V2-based feature extractor for facial expression analysis. This extractor operates on long sequences of frames extracted from videos to capture spatiotemporal features in facial expressions better. For physiological signal processing, we innovatively propose a “Local-Medium-Global” hierarchical feature extraction framework. This framework synergistically captures transient local details, rhythmic mid-range patterns, and global temporal dynamics within physiological signals, significantly reducing computational complexity while maintaining performance. Crucially, at the feature fusion stage, we introduce a Gated Attention Mechanism. This mechanism dynamically learns complex nonlinear inter-modal interactions, enabling adaptive deep synergistic fusion of cross-modal features, thereby driving substantial improvements in recognition performance.

In summary, this paper makes three core contributions:

- To address the inefficient modeling of coupled spatio-temporal features in continuous facial expression sequences, we introduce a computationally efficient synergistic architecture combining ConvNeXt V2 and lightweight Transformers for efficient spatio-temporal dynamic feature extraction.
- To overcome the challenge of unified modeling for multi-scale temporal patterns in physiological signals (transient local, rhythmic mid-range, and global dependencies), we develop a novel three-level hybrid feature extraction framework (“Local-Medium-Global”). This framework ensures computational efficiency while comprehensively capturing cross-scale bio-features.
- To mitigate the limitations of simple feature concatenation, such as modal redundancy and lack of complementarity, we propose a feature fusion module based on a Gated Attention Mechanism. This module adaptively learns and modulates the contribution weights of features from different modalities, enabling deep interaction and optimal collaboration at the feature level, effectively overcoming the drawbacks of naive concatenation.

In summary, the GBV-Net framework’s main innovation is its holistic, biologically inspired approach to multimodal emotion recognition. Unlike prior works, which often focus on shallow fusion or process modalities in isolation, GBV-Net introduces three synergistic innovations: (1) a computationally efficient spatio-temporal architecture for facial movements, (2) a hierarchical feature extractor for multi-scale physiological signal processing, and (3) a dynamic gated attention mechanism for adaptive feature-level fusion. The combination of specialized feature extractors and an intelligent fusion strategy is a significant evolution from existing methods.

The structure of the subsequent sections of this paper is as follows: Section 2 discusses the latest methods for extracting features from facial information and physiological signals (especially electroencephalograms) as a means of multimodal emotion recognition. Section 3 provides a detailed introduction to GBV-Net, a hierarchical fusion multimodal emotion recognition model based on facial expressions and physiological signals. Section 4 systematically describes the experimental framework, the datasets utilized, and the evaluation metrics employed, presents the results, and provides comparative analyses against existing methods. Finally, Section 5 summarizes the work, accompanied by a discourse on prospective avenues for future research.

## 2. Related Work

Emotion recognition holds significant value for diverse applications, including human-computer interaction (HCI) and mental health assessment. This importance has motivated substantial research interest in recent years. Consequently, the field has established itself as a systematic research domain. From a technical implementation perspective, emotion recognition systems based on deep learning are categorised primarily into two types according to the modality of the input data: unimodal and multimodal.

### 2.1. Unimodal Emotion Recognition

Unimodal emotion recognition employs a solitary data modality, encompassing facial expressions, physiological signals, text, or speech data. However, due to the susceptibility of unimodal data to noise and the inherent complexity of emotion recognition, the dependability and authenticity of results derived from models based solely on unimodal data are frequently questioned.

#### 2.1.1. Emotion Recognition from Facial Expressions

Facial expressions serve as a spontaneous and inherent manifestation of an individual’s psychological disposition, conveying a complex array of emotional information. Facial Expression Recognition (FER) aims to infer emotional states by analyzing facial expressions in multimedia data like images and videos. Driven by advances in multimedia technology, FER has become a prominent area of research focus in the fields of computer vision and artificial intelligence due to its broad application prospects. Meena et al. [7] developed a CNN solution capable of handling large-scale signal data. Their optimization strategy employed larger batch sizes, increased convolutional layer depth, and extended training epochs to enhance model performance. Similarly, focusing on architectural innovation, Chowdary et al. [8] systematically evaluated four transfer learning frameworks. By removing the fully connected layers of previously trained CNNs and reconstructing task-specific FC layers, they achieved an average recognition accuracy of 96% on 918 images from the Cohn-Kanade (CK+) database. Expanding application scenarios further, Minaee et al. [9] addressed challenges in FER, notably high intra-class variance and the poor generalization of traditional handcrafted features, by proposing an attention-based convolutional network model. Their method, which focuses on key facial regions, significantly outperformed existing models on four benchmark datasets, including FER-2013. Innovatively, they combined visualization techniques to reveal facial regions sensitive to different emotions. This end-to-end framework effectively overcame challenges like partial occlusion and image variations, offering a new approach for expression recognition in complex scenarios.

#### 2.1.2. Emotion Recognition from Physiological Signals

Compared to facial expressions, the core advantage of physiological signals lies in their authenticity and resistance to voluntary control, enabling a more objective assessment of emotional states. Recent research has primarily focused on EEG signals due to their direct reflection of central neural activity and high temporal resolution, which are crucial for capturing rapid emotional dynamics. Alongside EEG, other physiological signals such as EMG, ECG, and GSR also provide valuable insights from the peripheral nervous system, offering complementary information on emotional arousal and valence. For instance, ECG reflects heart rate variability linked to autonomic nervous system activity, while GSR indicates sympathetic arousal related to emotional intensity. Although EEG is often the primary focus in multimodal studies because of its rich cortical information, the integration of complementary modalities like ECG and GSR can enhance the robustness of emotion recognition systems. This body of work has yielded favorable results, showing the importance of physiological signals in affective computing. Zhu et al. [10] extracted Differential Entropy (DE) features from EEG signals, employed a Linear Dynamic System (LDS) for feature smoothing, and ultimately used a Support Vector Machine (SVM) for classification. Bhatti et al. [11] extracted time-domain and frequency-domain features from EEG signals and fed them directly into a classifier for emotion recognition. Algarni et al. [12] proposed a system framework aimed at enhancing the reliability of emotion recognition results to support precise medical decision-making. The framework’s initial phase involved the extraction of wavelet features, the Hurst exponent, and statistical features from EEG signals. Subsequently, a Binary Grey Wolf Optimization (BGWO) algorithm is employed for feature selection to identify the most discriminative patterns. Finally, a stacked Bidirectional Long Short-Term Memory (Bi-LSTM) network was utilized for emotion classification based on the selected features.

#### 2.1.3. Comparison Between Facial Expression- and Physiological Signal-Based Emotion Recognition

Emotion recognition from facial expressions and physiological signals differs in several key aspects. Facial expressions are external, voluntary or involuntary behavioral cues that cameras can easily capture, but they can also be consciously suppressed or fabricated. In contrast, physiological signals are internal, involuntary representations of autonomic nervous system activity. They are considered more objective and resistant to deliberate manipulation. Thus, they potentially provide a more reliable indicator of genuine emotional states. However, their acquisition requires specialized hardware and is more intrusive. This inherent complementarity is the core rationale for their fusion.

### 2.2. Multimodal Emotion Recognition

In recent years, multimodal emotion recognition has attracted significant research interest. The integration of physiological signals, particularly EEG, with facial expression features has become an increasingly explored subject in research. This fusion method utilizes complementary information from both modalities. Combining these features provides a more comprehensive characterization of emotional states. Consequently, recognition performance improves substantially. Salama et al. [13]. implemented this approach by converting brief EEG data into three-dimensional blocks. These blocks were then combined with synchronized sequences of facial images within corresponding temporal windows. Siddharth et al. [14] extracted features from facial image sequences, EEG signals, and peripheral physiological signals (e.g., ECG and GSR), achieving feature-level fusion through vector concatenation. Huang et al. [15] employed Adaptive Boosting (Adaboost) combined with a decision-level fusion strategy to integrate facial and EEG modality information, resulting in improved recognition accuracy. Xiang et al. [16] elicited emotions in subjects, simultaneously collected facial expression videos and physiological signals, and designed a Spatiotemporal Convolutional Neural Network (Spatiotemporal CNN) to analyze the performance of different modalities in emotion recognition.

However, despite the potential of multimodal fusion to enhance accuracy, current mainstream methods exhibit significant limitations in their feature fusion strategies. Existing approaches predominantly rely on simplistic linear weighting or feature concatenation [17], failing to deeply explore and model the potential complex nonlinear correlations and complementarities between features from different modalities. This shallow fusion mechanism struggles to fully exploit inter-modal synergies, limiting further improvements in model performance.

To address the challenge of feature fusion, this paper proposes an efficient method based on a gated attention mechanism. It aims to explicitly model and enhance the intrinsic relationships between multimodal information, thereby driving substantial improvements in multimodal emotion recognition performance. Specifically, we propose a model based on a modified ConvNeXt V2 architecture incorporating lightweight Transformers, designed to extract robust spatio-temporal dynamic features from facial image sequences. Concurrently, we design an innovative three-tier hybrid feature extraction framework (“Local-Medium-Global”) to efficiently capture fine-grained local patterns, mid-range rhythmic regularities, and global temporal dependencies within multimodal physiological signals. Finally, at the feature level, we introduce a Gated Attention Mechanism to perform adaptive deep fusion of the extracted facial and physiological features, fully mining their intrinsic relationships. The resulting fused features are then fed into a classifier to complete the emotion recognition task.

## 3. Methodology

### 3.1. GBV-Net Architecture Overview

**Figure 1:**
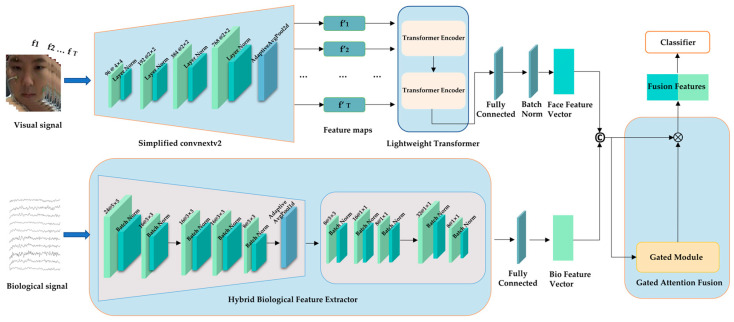
The overall architecture of the proposed Gated Biological Visual Network (GBV-Net).

Figure 1 shows the Gated Biological Visual Network multimodal emotion recognition model proposed in this paper. The model processes aligned temporal sequences of facial expression frames (visual stream) and physiological signals, including EEG, ECG, EOG, and GSR (biological stream). Its core comprises dual specialized extractors: a visual feature extractor based on an enhanced ConvNeXt V2 network that captures spatial features, followed by a lightweight Transformer encoder for modeling temporal dynamics in facial expressions, and a hybrid physiological feature extractor that employs a hierarchical structure combining 1D-CNNs for local patterns, a Temporal Convolutional Network (TCN) for medium-range dependencies, and a convolutional self-attention mechanism for global context. An innovative gated attention fusion module integrates the extracted features from both modalities. The resulting representation is ultimately classified to output emotion probabilities. This end-to-end architecture optimizes multimodal feature representation while ensuring computational efficiency.

**Detailed Architecture of the Gate-Based Bio-Visual Network (GBV-Net).** Visual streams process facial frame sequences through an enhanced ConvNeXt V2 (4 stages, with channel dimensions of 96, 192, 384, and 768) and a 2-layer Transformer (input: T × 3 × 224 × 224), yielding feature vectors of 128 dimensions. The physiological stream processes signal sequences through a 1D convolutional neural network (convolution kernel size 5 × 3), a spatiotemporal convolutional neural network (3 layers, dilation rates 1, 2, and 4), and a convolutional self-attention mechanism, yielding a feature vector of dimension 256. The gated fusion module combines these features via element-wise weighted averaging. The channels and convolutions have been clearly labeled in the diagram.

### 3.2. Multimodal Feature Extraction

This section describes methods for extracting features from visual signals and physiological signals. For visual signals, an improved ConvNeXt V2 architecture is employed, extracting static features through four levels of spatial downsampling and capturing temporal dynamics using a two-layer Transformer. Physiological signal processing uses a hybrid architecture that combines multi-scale 1D convolution, temporal convolution, and convolutional self-attention mechanisms to extract feature sequences. These are ultimately output as deep representations through a feature integration layer.

#### 3.2.1. Facial Feature Extraction

For facial features, the present study proposes a facial expression feature extraction architecture. By leveraging a modified ConvNeXt V2 architecture [18] and a lightweight Transformer temporal modeling module [19], it achieves joint modeling of spatial features and temporal dynamic features. Compared to the original ConvNeXt V2 architecture, our modified version implements three key adaptations for facial feature extraction: simplified stage configuration with stride and kernel adjustments (employing a 4 × 4 kernel in the initial layer instead of the standard 7 × 7 patch embedding), progressive channel reduction from (128, 256, 512, 1024) to (96, 192, 384, 768), and layer normalization placement optimized for video sequence processing. These architectural changes reduce model complexity while maintaining robust temporal feature extraction capabilities. This architecture divides facial feature extraction into two consecutive processing stages, spatial feature extraction using the modified ConvNeXt V2 and temporal dynamic modeling using the Transformer, significantly enhancing computational efficiency while ensuring feature discriminability.

In the spatial feature extraction stage, a modified ConvNeXt V2 architecture is employed for multi-level feature extraction. This module first employs a 4 × 4 convolutional layer with a stride of 4 on the input image to reduce it to a low-resolution feature space. The convolutional operation is expressed as follows:

(1) $$Y\left(i,j\right)=\sum_{m=0}^{k-1}\sum_{n=0}^{k-1}W\left(m,n\right)\cdot X\left(i+m,j+n\right)+b$$

In which X stands for the input facial image feature map, W is the convolution kernel of size K × K, b indicates the bias, i and j denote the spatial coordinates of the feature map, and Y represents the output feature map. The symbol in the equation denotes scalar multiplication between the kernel weight and the corresponding input value.

Subsequently, we perform feature transformation and dimensionality enhancement through a series of modular components consisting of convolutional layers, Layer Normalization (LayerNorm), and the GELU activation function. Compared to the original ConvNeXt V2, we simplified the network’s depth and width while retaining its efficient feature extraction capability. This architecture employs a layer-wise, dimension-increasing design that enables the network to capture multi-scale facial features, from local details to global semantics, at different hierarchical levels. The introduction of a lightweight Transformer module was made for the purpose of modeling temporal dependencies within the expression sequence, given the dynamic evolution of facial expressions over time. This module consists of a 2-layer Transformer encoder, where each encoder layer incorporates a multi-head self-attention mechanism and a feedforward neural network. The multi-head self-attention mechanism is shown below:

(2) $$MultiHead\left(Q,K,V\right)=Concat\left({\mathrm{head}}_{1},{\mathrm{head}}_{2},\ldots ,{\mathrm{head}}_{h}\right)\cdot W^{O}$$

In this context, Q, K, and V in ${\mathrm{head}}_{i}=\mathrm{Attention}\left(Q\cdot W_{i}^{Q},K\cdot W_{i}^{K},V\cdot W_{i}^{V}\right)$ represent the query, key, and value matrices, respectively. All of the learnable parameters $W_{i}^{Q},\;W_{i}^{K},\;W_{i}^{V},\;W^{O}$ are matrices, each of which has several attention heads, denoted by h.

The Transformer’s input is the feature sequence processed by the spatial feature extractor. To satisfy the input requirements of the Transformer architecture, the feature sequence dimensionality is adjusted accordingly. The self-attention mechanism effectively models dependency relationships across different time steps. Compared to traditional recurrent neural networks, such as LSTMs, the Transformer can more effectively capture long-range temporal dependencies. Additionally, it supports parallel processing, which substantially enhances training efficiency.

#### 3.2.2. Physiological Signal Feature Extraction

The bio-signal feature extraction module proposed in this study adopts a hierarchical architecture. This design integrates local feature extraction, temporal dependency modeling, and global correlation learning. It effectively captures multi-scale features and dynamic patterns inherent in bio-signals. The module consists of three core components: a local feature extractor, a temporal convolutional network (TCN), and an efficient convolutional self-attention mechanism. These components collaborate to extract deep features from bio-signals.

The Local Feature Extractor employs a CNN architecture tailored to capture transient local patterns and high-frequency features in bio-signals. This sub-module utilizes a dual-layer 1D convolutional architecture [20]. The refinement of features is attained through a progressive reduction in feature channels and a decrease in convolutional kernel size across layers. Each layer incorporates batch normalization and ReLU activation functions. These functions accelerate training convergence and enhance the model’s nonlinear expressive capacity. The local features are as follows:

(3) $$F_{\mathrm{local}}\left(X\right)=\mathrm{ReLU}\left(\mathrm{BN}\left(W\;*\;X+b\right)\right)$$

TCN [21] captures medium-length temporal dependencies in biological signals. The module consists of three dilated convolutional layers with progressively increasing dilation rates. By introducing gaps within the convolutional kernel, the receptive field expands exponentially. This expansion enables the extraction of dynamic features across multiple time scales. Each dilated convolution is followed by batch normalization and a ReLU activation function. The final layer reduces the feature dimension to eight. Medium-length feature extraction is represented as follows:

(4) $$F_{medium}\left(X\right)=\mathrm{ReLU}\left(\mathrm{BN}\left(\sum_{d\in \{1,2,4\}}W_{d}{\;*}_{d}X+b_{d}\right)\right)$$

in which d is the expansion rate and Wd is the weight. By adjusting the expansion rate, the TCN can effectively model medium-range dependencies in signals without increasing parameters and computation.

For the global dependency modeling stage in bio-signal feature extraction, we employ an efficient convolutional self-attention mechanism. This module first extracts local feature patterns through depthwise convolution operations. Subsequently, pointwise convolution adjusts channel dimensionality to capture richer feature representations. Building upon these features, a self-attention mechanism is subsequently delineated as a means to model long-range dependencies among features, thereby enabling the model to adaptively focus on salient discriminative segments within the signal sequence. Finally, feature transformation is performed via a lightweight feedforward network, and residual connections are incorporated to further enhance feature flow and gradient propagation. This design ensures computational efficiency and representational capacity while capturing global dependencies. The architecture effectively strikes a balance between model complexity and performance, making it particularly well-suited for processing long-sequence bio-signal data. Long-distance global associations are as follows:

(5) $$F_{\mathrm{global}}\left(X\right)=\mathrm{Residual}\left(\mathrm{FFN}\left(\mathrm{Attention}\left({\mathrm{Conv}}_{\mathrm{point}}\left({\mathrm{Conv}}_{\mathrm{depth}}\left(X\right)\right)\right)\right),X\right)$$

In this formulation, Convdepth and Convpoint represent depth-wise and point-wise convolution operations, respectively. Attention is indicative of the incorporated self-attention mechanism. FFN is an acronym for feedforward network, and Residual signifies the residual connection.

### 3.3. Feature Fusion

According to the latest findings in the neurosciences, the processing of emotions in humans is supported by a distributed network involving coordinated activity across multiple brain regions [22]. This network comprises several key nodes, including the occipitotemporal neocortex, which facilitates visual integration; the amygdala, which processes affective evaluations; the orbitofrontal cortex, which governs value-based decision-making; and the right frontoparietal cortex, which regulates spatial attention [23]. During the process of emotional regulation, the brain concurrently processes multisource heterogeneous physiological and visual signals [24]. Consequently, computational models that can effectively integrate multimodal features provide a more biologically plausible approach, aligning with the neurophysiological mechanisms underlying emotion generation.

The fusion module proposed in this study employs a gated attention fusion strategy, with the objective of achieving adaptive integration of facial expression and bio-signal attributes. The core design of the fusion module aims to dynamically balance the contribution weights of features from different modalities, effectively addressing the issues of complementarity and redundancy inherent in multimodal data. Specifically, a simplified ConvNeXt V2 network is initially employed to derive high-level semantic features of facial expressions, while a hybrid bio-feature extractor captures dynamic features from bio-signals. To avoid information redundancy caused by simple feature concatenation, the model incorporates a gating mechanism for fine-grained regulation of the fusion process. The combined facial and bio-signal feature vectors pass through a gating mechanism, utilizing a stack of fully connected layers with Sigmoid-based activation for multimodal fusion. This unit generates a weight vector matching the dimensionality of the input features, enabling dynamic weighting of features from disparate analytical modalities.

The primary advantage of the proposed Gated Attention Mechanism over traditional fusion strategies, such as simple concatenation or weighted averaging, lies in its adaptive and dynamic nature. Unlike static methods that apply fixed fusion rules, our gating unit learns to assign optimal, input-specific contribution weights to features from each modality. This core functionality enables two key benefits: firstly, it enhances robustness by automatically amplifying the influence of high-quality, discriminative features while suppressing those that are noisy or uninformative for a given sample; secondly, it effectively mitigates issues of modal redundancy and lack of complementarity inherent in naive fusion by modeling complex, non-linear inter-modal relationships. This dynamic, feature-aware integration effectively utilizes multimodal information, which is the cornerstone of the improved emotion recognition performance demonstrated by our experiments. The fusion part is shown below:

(6) $$F_{\mathrm{fused}}=\left[F_{\mathrm{face}},F_{\mathrm{bio}}\right]⊙\sigma \left(\left[F_{\mathrm{face}},F_{\mathrm{bio}}\right]\right)$$

In this case, F_face_ and F_bio_ represent facial features and biometric features, respectively, while F_fused_ represents the fused features where the operator ⊙ denotes the element-wise multiplication (Hadamard product).

## 4. Experimental Results and Analysis

Two publicly available benchmark datasets, DEAP [25] and MAHNOB-HCI [26], are employed for model validation in this study. Both datasets provide multimodal physiological signals and facial expression videos recorded simultaneously, offering standardized evaluation environments for multimodal emotion recognition research. Experiments integrate nearly complete multimodal data from all available participants (after invalid samples are removed) to ensure the statistical significance of the evaluation results. Model performance was assessed using a 10-fold cross-validation strategy. This method involves the random partitioning of the dataset into ten mutually exclusive subsets. In this particular instance, the training process involves the sequential utilization of nine distinct subsets. Concurrently, the residual subset functions as the designated test set, thereby ensuring the systematic exploration of all ten combinations. The final performance metrics represent the average values across all ten test iterations. The calculation formula is as follows:

(7) $$Acc_{avg}=\frac{1}{10}\sum_{k=1}^{10}Acc_{k}$$

Here, $Acc_{avg}$ represents the accuracy rate of the k-fold validation. This design effectively reduces the impact of random data partitioning on the results, providing a more objective reflection of the model’s generalization ability.

### 4.1. Experimental Dataset and Preprocessing

The DEAP dataset is a multimodal database designed for studying human emotional states. It contains synchronized recordings from 32 participants exposed to 40 emotion-eliciting video clips (each 63 s), capturing central neural system signals as indicated by EEG, EMG, and GSR measures, as well as peripheral physiological signals, and facial expression video streams. For each stimulus presented, participants evaluated their responses along the dimensions of Valence, Arousal, Dominance, Liking, and Familiarity. EEG signals in DEAP were downsampled. Initially, the signals were sampled at a rate of 128 hertz. Then, they underwent a bandpass filtering procedure, during which the frequencies were limited to a range between 4.0 and 45 Hz and processed with blind source separation to remove ocular artifacts. Detailed specifications are provided in Table 1.

The MAHNOB-HCI database is another multimodal emotional database comprising recordings of 30 participants across 20 experimental sessions. It synchronously captures facial videos and central nervous system signals, peripheral physiological signals, and eye movement data. Notably, stimulus durations vary across trials, requiring precise segmentation of valid time windows based on official annotation files. Emotional annotations utilize the following four dimensions: valence, arousal, control, and predictability. However, the integrity of the data from three participants was compromised, resulting in their exclusion from the study. Consequently, the analysis was based on the data from 27 participants, thereby ensuring the reliability and validity of the study’s findings. Complete dataset characteristics are summarized in Table 1.

The data preprocessing methodology employed in this study is detailed below: For facial expression data, we performed temporal sampling at 10 fps for DEAP and 12 fps for MAHNOB-HCI to sufficiently capture facial dynamics, with extracted frames undergoing pose-normalized alignment using 68 facial landmarks detection [27], followed by facial region cropping to preserve expression-critical features. For biosensor data, signals were downsampled to 128 Hz, bandpass-filtered, segmented using non-overlapping 1 s windows, and baseline-corrected by subtracting mean baseline values to mitigate signal drift. Regarding data augmentation, domain-appropriate techniques were employed on facial images, including horizontal flipping, color jittering, and Gaussian blurring, distinct from augmentation methods in fields like remote sensing [28], while additive noise, temporal shifting, and amplitude scaling were applied to bio-signals. Notably for EEG signals, both datasets share identical channel configurations and electrode placements (Table 2), ensuring consistent neurophysiological feature extraction.

The evaluation of GBV-Net employed a 10-fold cross-validation strategy on each dataset (DEAP and MAHNOB-HCI) rather than a single separate test set. This approach is widely adopted in affective computing research due to the constrained size of publicly available multimodal datasets. It maximizes the use of available data for both training and validation, providing a statistically robust performance estimate that mitigates the variance associated with a single random train-test split.

**Figure 2:**
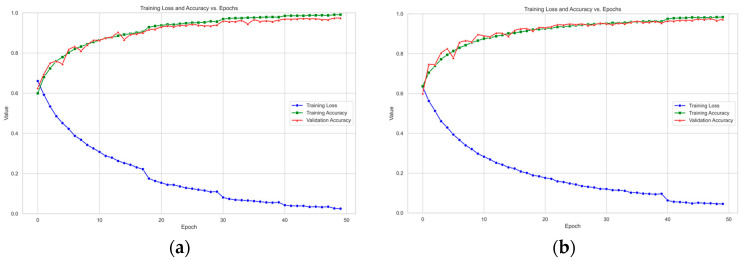
Model performance evaluation curves in the DEAP dataset. Here, (**a**) represents the Valence dimension curve and (**b**) represents the Arousal dimension curve.

**Figure 3:**
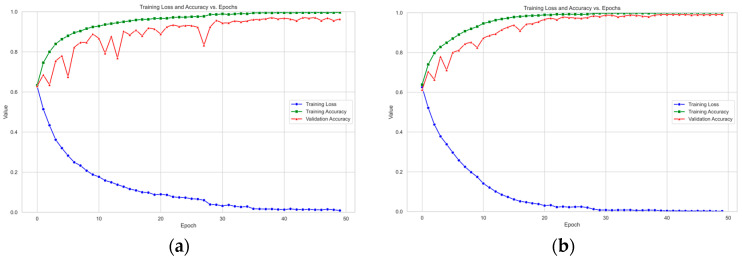
Model performance evaluation curves in the MAHNOB-HCI dataset. Here, (**a**) represents the Valence dimension curve and (**b**) represents the Arousal dimension curve.

To rigorously address potential overfitting and ensure the validity of the cross-validation results, we implemented several measures:

- Data Augmentation: As detailed in Section 4.1, extensive data augmentation techniques (e.g., horizontal flipping and color jittering for faces; additive noise and temporal shifting for bio-signals) were applied during training. This increases the diversity of the training data, forcing the model to learn more generalized features.
- Monitoring Learning Curves: The training and validation accuracy and loss curves were meticulously monitored throughout the training process (as shown in Figure 2 and Figure 3). The close alignment and concurrent convergence of these curves, without a significant divergence, indicate that the model was learning generalizable patterns rather than memorizing the training data.
- Regularization Techniques: The model architecture inherently incorporates modern regularization techniques, such as Batch Normalization and residual connections, which help stabilize training and reduce overfitting.

While cross-validation provides a strong indication of model performance on data from a similar distribution, we acknowledge that the ultimate test of generalizability involves evaluation on a completely independent dataset. This remains a focus for our future work.

### 4.2. Main Results and Comparative Analysis

All experiments were conducted on a server running the Windows 10 Professional operating system. The hardware configuration comprised an Intel(R) Xeon(R) Silver 4210R CPU @ 2.40 GHz and an NVIDIA RTX A6000 graphics card with 48 GB of VRAM. The proposed model was implemented using the PyTorch 1.13.0 framework with CUDA 12.6 support. To optimize the hyperparameter settings, the batch size was set to 256, and the learning rate was set to 0.001. During training, the Adam algorithm was used in conjunction with an optimizer, and binary classification cross-entropy was used as the loss function.

Figure 2 and Figure 3 show the trends in training accuracy, validation accuracy, and training loss during the training process of the model proposed in this paper on the DEAP and MAHNOB-HCI datasets.

As shown in Figure 2 and Figure 3, the training loss on the DEAP dataset consistently decreases with increasing iterations and eventually plateaus. This indicates that the model effectively learns data patterns and optimizes its parameters during training. Concurrently, the training accuracy exhibits a steady rise. The validation accuracy also demonstrates an overall upward trend, maintaining close alignment with the training accuracy curve. The model exhibits remarkable generalization on the DEAP dataset, as evidenced by the tight agreement between training and validation results. On the MAHNOB-HCI dataset, the training loss similarly exhibits a continuous decline, accompanied by a consistent improvement in training accuracy. Notably, despite some fluctuations in validation accuracy (Figure 3a) attributed to the dataset’s more complex and heterogeneous sample distribution, the overall trend remains upward. Furthermore, the validation accuracy eventually converges towards the training accuracy. This observation demonstrates the model’s effectiveness in identifying salient emotional features and its adaptability to the challenging demands of complex datasets.

A comparative analysis of the learning curves from the DEAP and MAHNOB-HCI datasets reveals distinctive patterns. The smoother curves observed in the DEAP dataset suggest a more homogeneous data distribution, resulting in more stable model convergence. In contrast, fluctuations in the validation accuracy on the MAHNOB-HCI dataset reflect its higher inherent data complexity. Notably, these variations also demonstrate the strong robustness of GBV-Net in handling challenging and heterogeneous scenarios.

The classification accuracy of the proposed model is shown in Table 3.

The model demonstrates notable efficacy in binary classification tasks when evaluated on the DEAP dataset. Specifically, the model achieves an accuracy of 94.68% for valence and 95.93% for arousal recognition. Notably, on the MAHNOB-HCI dataset, the model attains even higher accuracies of 97.48% for valence and 97.78% for arousal in the corresponding binary classification tasks. These results not only demonstrate a significant advantage over the accuracies reported for other existing methods listed in the table but also exhibit superior and consistent performance across both datasets and emotional dimensions. This provides robust evidence for the effectiveness and strong generalization capability of the proposed model.

To evaluate our model’s classification performance, we benchmarked it against leading multimodal emotion recognition approaches. All comparative results are provided in Table 3. Yuvaraj et al. [29] systematically evaluated various classical EEG features, including fractal dimension (FD) and Hjorth parameters, establishing the significance of feature engineering in identifying valence and arousal dimensions. Meanwhile, Huang [15] proposed a multimodal emotion recognition framework integrating facial expressions and EEG, while Li et al. [30] developed MindLink-Eumpy, an open-source toolkit for multimodal emotion recognition. These works, from the perspectives of framework design and tool implementation, respectively, validated the feasibility of significantly enhancing recognition performance through decision-level fusion strategies, offering promising approaches to overcome the limitations of unimodal methods. Furthermore, Zhang et al. [31] introduced a hierarchical self-attention-based framework for spatiotemporal modeling, demonstrating its potential to effectively capture long-range dependencies and critical spatial information within EEG signals for improved recognition accuracy. Siddharth et al. [14] explored the use of deep networks for processing transformed physiological signal features and multi-modal fusion, representing a trend towards deep learning advancements in this field.

In addition to accuracy, we evaluated GBV-Net using precision, recall, and F1-score to provide a comprehensive assessment. As shown in Table 4, the model achieves exceptionally high and balanced performance across all metrics on both datasets. The F1-scores, which harmonize precision and recall, are particularly strong, reaching up to 96.35% for Arousal on DEAP and 97.74% for Arousal on MAHNOB-HCI. While most metrics are closely aligned, the model exhibits a slight but consistent tendency towards higher recall compared to precision (e.g., Arousal on DEAP: Recall 96.82% vs. Precision 95.89%). The model is slightly better at identifying true positive samples, with only a slight drop in precision. The high values and agreement across metrics confirm the model’s robustness and balanced classification capability for emotional dimensions.

Based on the research and analysis of the aforementioned classical methods, the GBV-Net framework presented in this paper is a significant advancement in multimodal emotion recognition. Its superior performance is evident in Table 3. This advancement stems from three key innovations: (1) a dynamic gated attention mechanism that learns intermodal relationships adaptively, overcoming the limitations of the static fusion schemes used in previous studies, (2) a hierarchical feature extraction design that captures multiscale temporal patterns more effectively than standard approaches, and (3) an end-to-end trainable framework that optimally integrates complementary multimodal information. In contrast to the hierarchical self-attention mechanism used by Zhang et al. [31], our framework uses a spatiotemporal feature extraction architecture that combines ConvNeXt V2 and Transformer. Unlike the static fusion strategies adopted by Huang [15] and Li et al. [30] for multimodal data, the present study introduces a dynamic gated attention mechanism. This mechanism facilitates the integration of facial expressions and physiological signals through a learnable feature weighting process. Departing from the classical feature engineering paradigm explored by Yuvaraj et al. [29] and the PSD heatmap transformation method used by Siddharth et al. [14] for physiological signal processing, GBV-Net constructs a three-stage processing pipeline: local convolution, temporal modeling, and convolutional self-attention. This pipeline implements true end-to-end deep feature learning. Additionally, the framework incorporates techniques such as adaptive pooling, residual connections, and depthwise separable convolutions. These components collectively enhance the model’s adaptability to long sequences and computational efficiency. Experimental results demonstrate that this framework surpasses the aforementioned related studies on classification tasks using both the DEAP and MAHNOB-HCI datasets, offering a superior solution for multimodal emotion recognition.

### 4.3. Ablation Experiment

**Figure 4:**
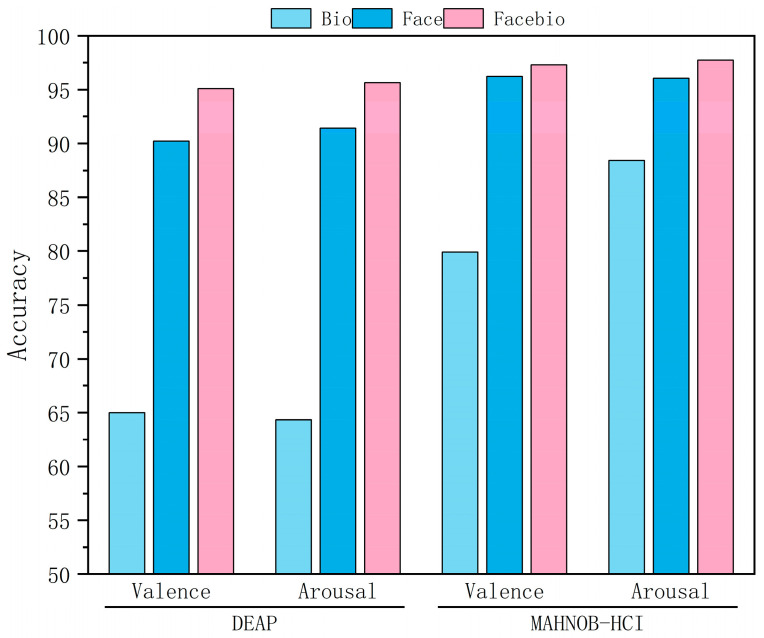
Classification results of ablation experiments (Accuracy).

**Figure 5:**
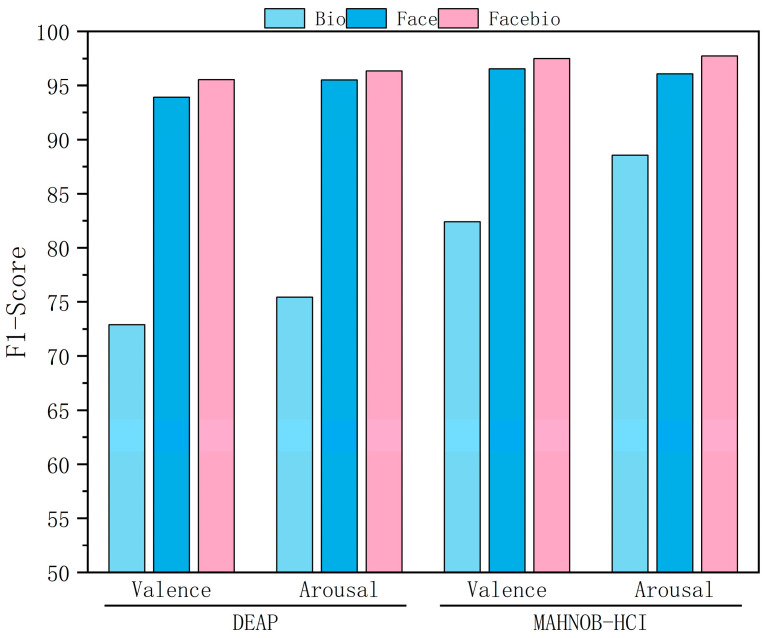
Classification results of ablation experiments (F1-Score).

To investigate the superiority of multimodal over unimodal emotion recognition, we conducted a systematic validation study across two datasets. The results are presented in Table 5, which shows the detailed accuracy and F1-score, and are visualized in Figure 4 and Figure 5, which show the ablation results. The facial modality demonstrated significant advantages on the DEAP and MAHNOB-HCI datasets, achieving stable accuracies exceeding 90% and F1 scores above 93.9%. In contrast, the physiological modality exhibited relatively limited performance. Multimodal fusion consistently improved performance across both evaluation metrics. On the DEAP dataset, valence recognition accuracy improved by over 4 percentage points to 95.10%, and the F1-score reached 95.52%. Arousal recognition accuracy improved to 95.65%, and the F1-score reached 96.35%. On the MAHNOB-HCI dataset, the fused model achieved remarkable performance, exceeding 97.2% accuracy and 97.5% F1-score across both dimensions. Notably, performance gains for arousal consistently surpassed those for valence, indicating the unique value of physiological signals in capturing emotional intensity. While bio-signals showed limited accuracy of around 65% on DEAP, they achieved substantially higher F1-scores of 72.89% for valence and 75.42% for arousal, demonstrating their ability to handle class imbalance more effectively. The final fused model approached or surpassed 95% accuracy and F1-score on all four tasks, peaking at 97.73% and 97.74%, respectively, for arousal on MAHNOB-HCI. This robust, multi-metric performance validates that facial features provide foundational discriminative power, that physiological signals complement dynamic responses with improved class balance, and that the gating fusion mechanism effectively coordinates their strengths. Cross-dataset consistency further validates GBV-Net’s ability to generalize in the dynamic coordination of multimodal information.

### 4.4. Visual Analysis and Robustness Evaluation

**Figure 6:**
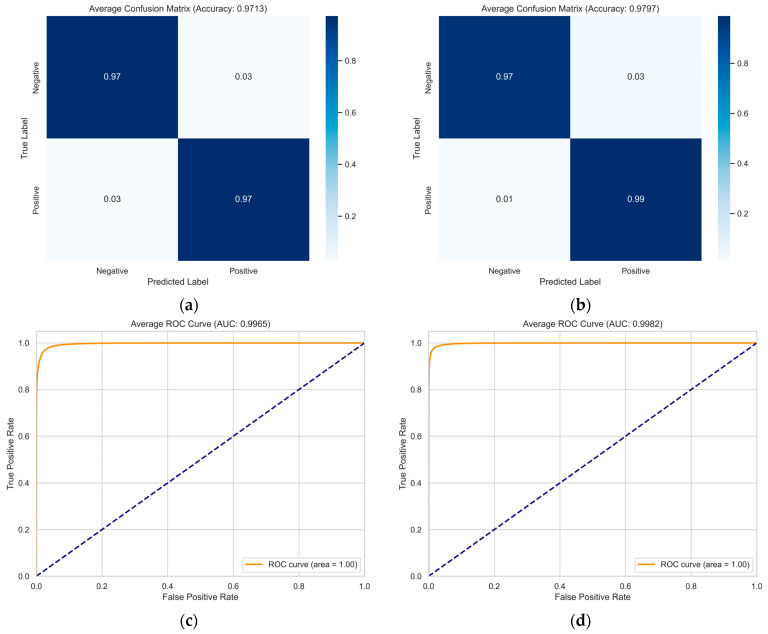
Classification average confusion matrix for the DEAP dataset on the (**a**) valence and (**b**) arousal dimensions ROC curves for the (**c**) valence and (**d**) arousal dimensions.

To further validate GBV-Net’s performance and comprehensively address potential overfitting concerns, we conducted extensive visual analysis on the test folds. Figure 6 presents the confusion matrices and ROC curves for valence and arousal classification on the DEAP dataset. The confusion matrices reveal exceptional classification performance, with diagonal values exceeding 97% for both valence (97.13%) and arousal (97.97%). More importantly, the misclassification rates are remarkably low, with false positive and false negative rates consistently below 3%, demonstrating the model’s balanced predictive capability across both classes.

**Figure 7:**
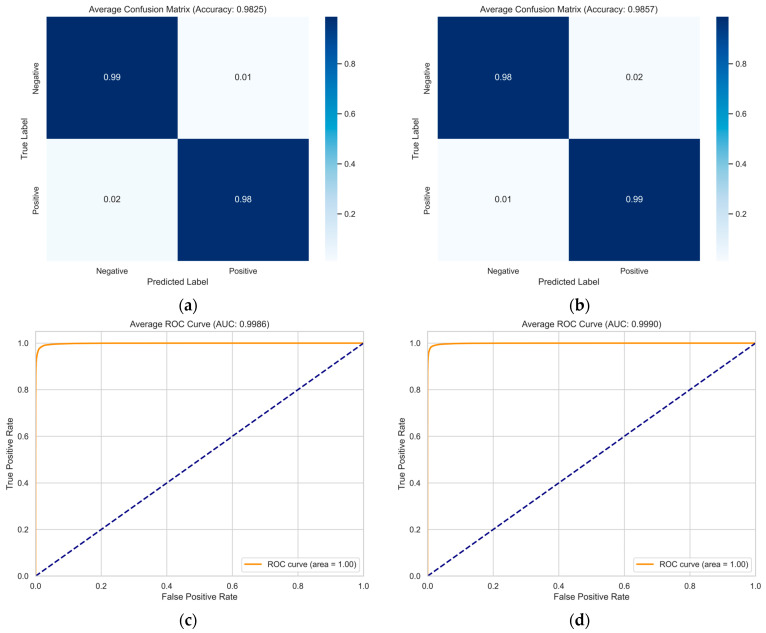
Classification average confusion matrix for the MAHNOB-HCI dataset on the (**a**) valence and (**b**) arousal dimensions ROC curves for the (**c**) valence and (**d**) arousal dimensions.

Figure 7 shows corresponding results for the MAHNOB-HCI dataset, where the model achieves even higher performance. The confusion matrices exhibit near-perfect classification, with accuracy reaching 98.25% for valence and 98.57% for arousal. The minimal off-diagonal values (all below 2%) indicate virtually no significant misclassification patterns.

The ROC curves provide further compelling evidence of the model’s discriminative power. GBV-Net achieves near-perfect AUC scores of 0.9965 (valence) and 0.9982 (arousal) on DEAP and 0.9986 (valence) and 0.9990 (arousal) on MAHNOB-HCI. All ROC curves approach the top left corner, indicating an ideal classification scenario. These exceptional AUC values, combined with the consistently high true positive rates and low false positive rates across both datasets, provide robust evidence that GBV-Net generalizes excellently without overfitting.

These comprehensive visualizations, when considered alongside our rigorous 10-fold cross-validation strategy and implemented anti-overfitting measures (including data augmentation, batch normalization, and residual connections), offer multi-faceted validation of the model’s robustness. The model maintains stellar performance across different data segments and exhibits reliable behavior characteristic of well-generalized systems.

## 5. Conclusions

The proposed framework, termed GBV-Net, is a pioneering multimodal emotion recognition system that integrates physiological signals and facial expressions synergistically. The model extracts discriminative features directly from raw physiological data and facial video streams. It employs a gated attention fusion mechanism to dynamically weight cross-modal interactions. In terms of facial expression feature extraction, the combination of an improved ConvNeXt V2 Tiny structure and a lightweight Transformer temporal modeling module enables joint modeling of spatial features and temporal dynamic features, thereby improving feature extraction capabilities and training efficiency. Physiological signal processing adopts a three-tier hierarchical feature abstraction framework, where cascaded convolutional blocks progressively capture local motifs, mid-range dependencies, and global contextual patterns. The gated cross-attention fusion module adaptively recalibrates modality-specific contributions, significantly boosting recognition robustness. The findings of the present study demonstrate that this method achieves a high level of accuracy in identifying emotions. Combining facial expressions and physiological signals yields a superior recognition effect compared to using a single modality alone. In terms of computational efficiency, the complete 10-fold cross-validation for both valence and arousal dimensions on the DEAP dataset required approximately 21 h, while on the MAHNOB-HCI dataset it took approximately 13 h. This difference is primarily attributed to the larger scale of the DEAP dataset (32 participants × 40 trials) compared to MAHNOB-HCI (27 participants × 20 trials). Crucially, the inference time for a single sample remained highly efficient at under 10 milliseconds for both datasets, demonstrating the model’s practical suitability for real-time emotion recognition applications. While the training phase demands substantial computational resources due to the deep multimodal architecture, the exceptional inference efficiency makes GBV-Net viable for deployment in real-world systems. To address the computational cost for resource-constrained environments, our immediate future work will prioritize model compression techniques, including neuron pruning and quantization, to significantly reduce computational requirements while maintaining performance levels. Subsequent extensions will incorporate additional modalities like speech and body gestures, with careful optimization of their computational overhead.

## Figures and Tables

**Table 1:** Dataset details.

Attribute	DEAP	MAHNOB-HCI
Subjects	32	27
Available channels	40	38
Length of each train	60 s	49 s–117 s
Trail of each subject	40	20
Emotional description	Valence, Arousal	Valence, Arousal

**Table 2:** Electroencephalogram signal electrode channel arrangement.

Channel Number	Channel Name	Channel Number	Channel Name
1	Fp1	17	Fp2
2	AF3	18	AF4
3	F3	19	Fz
4	F7	20	F4
5	FC5	21	F8
6	FC1	22	FC6
7	C3	23	FC2
8	T7	24	Cz
9	CP5	25	C4
10	CP1	26	T8
11	P3	27	CP6
12	P7	28	CP2
13	PO3	29	P4
14	O1	30	P8
15	Oz	31	PO4
16	Pz	32	O2

**Table 3:** Comparison of GBV-Net model classification results with existing methods.

Datasets	Authors	Accuracy	
		Valence	Arousal
DEAP	Yuvaraj et al. [29]	78.18%	79.90%
	Huang et al. [15]	80.30%	74.23%
	Li et al. [30]	71.00%	58.75%
	Zhang et al. [31]	72.89%	77.03%
	Siddharth et al. [14]	79.52%	78.34%
	Ours	95.10%	95.65%
MAHNOB-HCI	Yuvaraj et al. [29]	83.98%	85.58%
	Huang et al. [15]	75.21%	75.63%
	Li et al. [30]	70.04%	72.14%
	Zhang et al. [31]	79.90%	81.37%
	Siddharth et al. [14]	85.49%	82.93%
	Ours	97.28%	97.73%

**Table 4:** Comprehensive performance metrics of GBV-Net (%).

Datasets	Dimension	Accuracy	Precision	Recall	F1-Score
DEAP	Valence	95.10	95.21	95.84	95.52
	Arousal	95.65	95.89	96.82	96.35
MAHNOB-HCI	Valence	97.28	97.67	97.33	97.50
	Arousal	97.73	97.51	97.98	97.74

**Table 5:** Classification results of ablation experiments (%).

Datasets	Modal	Accuracy		F1-Score	
		Valence	Arousal	Valence	Arousal
DEAP	Bio	64.99	64.34	72.89	75.42
	Face	90.22	91.40	93.91	95.49
	Facebio	95.10	95.65	95.52	96.35
MAHNOB-HCI	Bio	79.89	88.42	82.40	88.55
	Face	96.23	96.05	96.55	96.08
	Facebio	97.28	97.73	97.50	97.74

## Data Availability

Data are contained within the article.

## Abbreviations

The following abbreviations are used in this manuscript:

EEG	Electroencephalogram
ECG	Electrocardiogram
EOG	Electrooculogram
GSR	Galvanic skin response
Bio	Biological
